# Emergence and competition of virus variants in respiratory viral infections

**DOI:** 10.3389/fimmu.2022.945228

**Published:** 2023-02-14

**Authors:** Nikolai Bessonov, Daria Neverova, Vladimir Popov, Vitaly Volpert

**Affiliations:** ^1^ Institute of Problems of Mechanical Engineering, Russian Academy of Sciences, Saint Petersburg, Russia; ^2^ S.M. Nikolskii Mathematical Institute, Peoples‘ Friendship University of Russia, Moscow, Russia; ^3^ Institut Camille Jordan, University Lyon, Villeurbanne, France

**Keywords:** viral infection, emergence of variants, immune escape, competition, reaction-diffusion equations

## Abstract

The emergence of new variants of concern (VOCs) of the SARS-CoV-2 infection is one of the main factors of epidemic progression. Their development can be characterized by three critical stages: virus mutation leading to the appearance of new viable variants; the competition of different variants leading to the production of a sufficiently large number of copies; and infection transmission between individuals and its spreading in the population. The first two stages take place at the individual level (infected individual), while the third one takes place at the population level with possible competition between different variants. This work is devoted to the mathematical modeling of the first two stages of this process: the emergence of new variants and their progression in the epithelial tissue with a possible competition between them. The emergence of new virus variants is modeled with non-local reaction–diffusion equations describing virus evolution and immune escape in the space of genotypes. The conditions of the emergence of new virus variants are determined by the mutation rate, the cross-reactivity of the immune response, and the rates of virus replication and death. Once different variants emerge, they spread in the infected tissue with a certain speed and viral load that can be determined through the parameters of the model. The competition of different variants for uninfected cells leads to the emergence of a single dominant variant and the elimination of the others due to competitive exclusion. The dominant variant is the one with the maximal individual spreading speed. Thus, the emergence of new variants at the individual level is determined by the immune escape and by the virus spreading speed in the infected tissue.

## 1 Introduction

Among many questions raised by the ongoing epidemic coronavirus disease of 2019 (COVID-19), the emergence of new variants of the SARS-CoV-2 infection is of primary importance. The emergence of a new variant of concern (VOC) can be considered as a stage process. First of all, mutations in the virus genome in an infected individual lead to the appearance of a viable variant distinct from the existing variant. Next, this new variant should replicate faster in the infected tissues in order to outcompete the previous variant and to produce a sufficient number of copies to be transmitted to other individuals. Finally, this new variant should have a larger transmission rate to spread in the population. In this work, we will develop a mathematical model describing the emergence of new variants/strains in an infected tissue and of the competition between the variants. Let us note that virus competition in tissue (culture) and in the population represent two different processes, and the conditions to win these competitions are also different. As it was recently shown, in the competition of two virus variants in cell culture, the variant with a larger individual spreading speed dominates and eliminates another one (1, 2). The competition of two virus variants in the presence of the immune response will be studied in this work, and the result of this competition will also be formulated in terms of individual spreading speeds. On the other hand, if two virus variants spread in the human population, the variant with a larger transmission rate spreads faster and eliminates another one (3), assuming that they are mutually exclusive due to the acquired immunity. Furthermore, the infection transmission rate for respiratory viral infections correlates with the viral load in the upper respiratory tract. Spreading speed and the viral load are different characteristics of viral infections, and they depend on cell types. As such, the Delta variant of the SARS-CoV-2 infection has larger spreading speed and outcompetes the Omega variant in the culture of human lung cells, while it is opposite in the culture of epithelial nasal cells (1, 4, 5). Moreover, the viral load of the Omicron variant is larger in the upper respiratory tract providing its faster transmission rate.

### 1.1 SARS-CoV-2 variants

SARS-CoV-2 genome is relatively large and contains approximately 30,000 bases that code for four structural proteins (spike, nucleoprotein, envelope, and membrane) and 16 non-structural proteins participating in virus multiplication and interfering with the immune response. Persistent COVID-19 pandemic provokes mutations and the emergence of numerous virus variants. Mutations in spike protein, responsible for virus binding to ACE2 membrane receptors appear to be most important in the regulation of SARS-CoV-2 pathogenicity. The first critical spike protein mutation was the substitution D614G that resulted in higher infectivity and promoted the spread of the D614G variant worldwide toward fixation in June 2020 (6).

Among numerous lineages that arise on the D614G variant, five of them have been classified as the variants of concern (7):

Alpha (B.1.1.7 lineage), characterized by nine spike protein mutations, was first collected in England in 20 September 2020 and started to spread rapidly in mid-October 2020 to constitute in January 2021 86% of all SARS-CoV-2 genomes that were sequenced in England. According to several studies, the Alpha variant had a high replicative advantage (estimates to be in range 1.43–2.18) with respect to pre-existing variants in the UK (8–10).

Beta (B.1.351 lineage), characterized by three spike protein mutations, was first collected in May 2020 in South Africa (11). Gamma (P.1 lineage) was characterized by nearly identical three mutations as Beta first collected in Brazil in November 2020. It was estimated that P.1 may be 1.7- to 2.4-fold more transmissible and that previous (non-P.1) infection (12).

Delta (B.1.617.2 lineage), characterized by two spike protein mutations, was first collected in October 2020 in India. It spread in India and then Europe outcompeting the Alpha variant. In an *in vitro* study, B.1.617.2 was found to be sixfold less sensitive to serumneutralizing antibodies from recovered individuals and eightfold less sensitive to vaccine-elicited antibodies as compared to the wild-type (WT) Wuhan-1 bearing D614G (13). This allowed Delta to spread in populations that were already exposed to earlier variants, and, as consequence, it constituted more than 97% of all SARS-CoV-2 genomes sequenced in October 2021 (based on Global Initiative on Sharing Avian Influenza Data (GISAID) database).

Omicron (Pango B.1.1.529 lineage) is characterized by multiple mutations in spike protein; three small deletions, one small insertion, and 30 substitutions with respect to the original variant. Of these changes, 15 are located in the receptor binding domain (residues 319–541) (14). It first spread in Gauteng province of South Africa growing with the doubling time of approximately 3–4 days and becoming dominant in the province by the end of November 2021 (15).

Variants Beta, Gamma, and Delta initiated rapid outbreaks, respectively, in South Africa, Brazil, and India. Mutations, which determined these three variants, are associated with reduction in neutralization by convalescent plasma and specific therapeutic antibodies. The existing vaccines, all developed on the basis of the wild-type SARS-CoV-2 strain, have smaller efficacy against these variants, and recovered individuals are prone to subsequent infections with the new variants (16).

### 1.2 Modeling of the emergence of new variants

Virus multiplication in an organism can be considered as competition between its replication and death. If the replication rate exceeds the death rate, the viral load grows providing infection progression. Both processes can depend on the virus genotype leading to the emergence and competition of virus quasi-species (17–20). As such, the behavior of SARS-CoV-2 quasi-species shows complex dynamics depending on time and the anatomical site (21). The time dynamics of HIV variants with their competitive exclusion or coexistence are studied in (19). Introducing viability intervals in the genotype space (or viability domains in the multidimensional space) where the total rate is positive, we associate different variants (strain and quasi-species) to different intervals (22, 23). If the original variant belongs to one of such intervals, new variants in other intervals can emerge due to random mutations (diffusion) in the genotype space. Changes in the virus genome due to mutations can be advantageous or disadvantageous with respect to its survival and multiplication. This mechanism of the emergence of new variants can be modeled with reaction–diffusion equations with space-dependent coefficients in the genotype space (24).

If we assume that mutations are neutral, that is, they do not give advantage in virus survival and replication, then, new variants can appear due to the immune escape. Namely, antigen-specific T- and B lymphocytes in the adaptive immune response stimulated by certain antigen are efficient in some areas around this antigen in the genotype space, but they lose their efficiency for distant antigens. Random mutations in the virus genome lead to the emergence of new variances outside of the area covered by the immune response.

In this work, we will consider the second mechanism where new virus variants appear due to the interaction of the cross-reactivity of the immune response and virus escape (Section 4). It can be modeled with non-local reaction–diffusion equations for the virus density distribution in the genotype space. Similar to the emergence of biological species, it is based on competition, reproduction, and mutations (25, 26). Compared to the previous studies, we will consider a more detailed and biologically realistic model including the concentrations of uninfected cells, infected cells, viruses and the immune response (Section 2).

Let us note that both mechanisms (genotype-dependent survival/replication rates and immune escape) can be considered together. We consider them separately in order to simplify the analysis and presentation. Moreover, a further investigation of the competition of different variants does not depend on the mechanism of their emergence.

### 1.3 Virus competition in cell culture and tissue

A viral infection spreads in cell culture as a reaction–diffusion wave (27–30). The wave speed is determined by the parameters of the model, such as the rate of cell infection, the replication rate, and the replication delay. The wave speed can differ for different variants. If two different variants are introduced in the same cell culture, then they begin to compete for uninfected cells. The results of modeling show that one of them outcompetes and eliminates another one. The condition of winning the competition can be formulated in terms of the wave speed: the variant with a larger individual wave speed wins the competition (1). Comparison with the experimental data on the time-dependent viral load for the Delta and Omicron variants of the SARS-CoV-2 infection (4, 5) allows the determination of the parameters of the model (1, 2). After that, we can compare the individual wave speeds of the two variants in different cell cultures and predict the winning variant. As such, the model predicts that the Delta variant wins the competition with the Omicron variant in the culture of lung cells and loses it in the culture of nasal epithelial cells (1). These conclusions are confirmed by modeling the competition of the two variants and by the experimental results on their competition (4).

In this work, we continue to investigate the competition of virus variants in the case of living tissue instead of cell cultures considered in the previous works. The main difference is in the presence of an adaptive immune response mediated by cytotoxic T lymphocytes killing infected cells and by neutralizing antibodies produced by B lymphocytes. Let us note that the antigen-specific adaptive immune response determines virus dynamics in the genotype space, while the non-specific innate immune response is less essential from the point of view of the immune escape and emergence of new variants. For this reason, and for the sake of simplicity, we restrict ourselves in this work to the adaptive immune response. Moreover, we will consider a single variable for the immune cells. The model is introduced in the next section. We will discuss the basic properties of infection progression in the host organism in Section 3. The emergence of new virus variants is studied in Section 4 and virus competition in Section 5. We conclude the paper with discussion.

## 2 Model formulation

**Figure 1:**
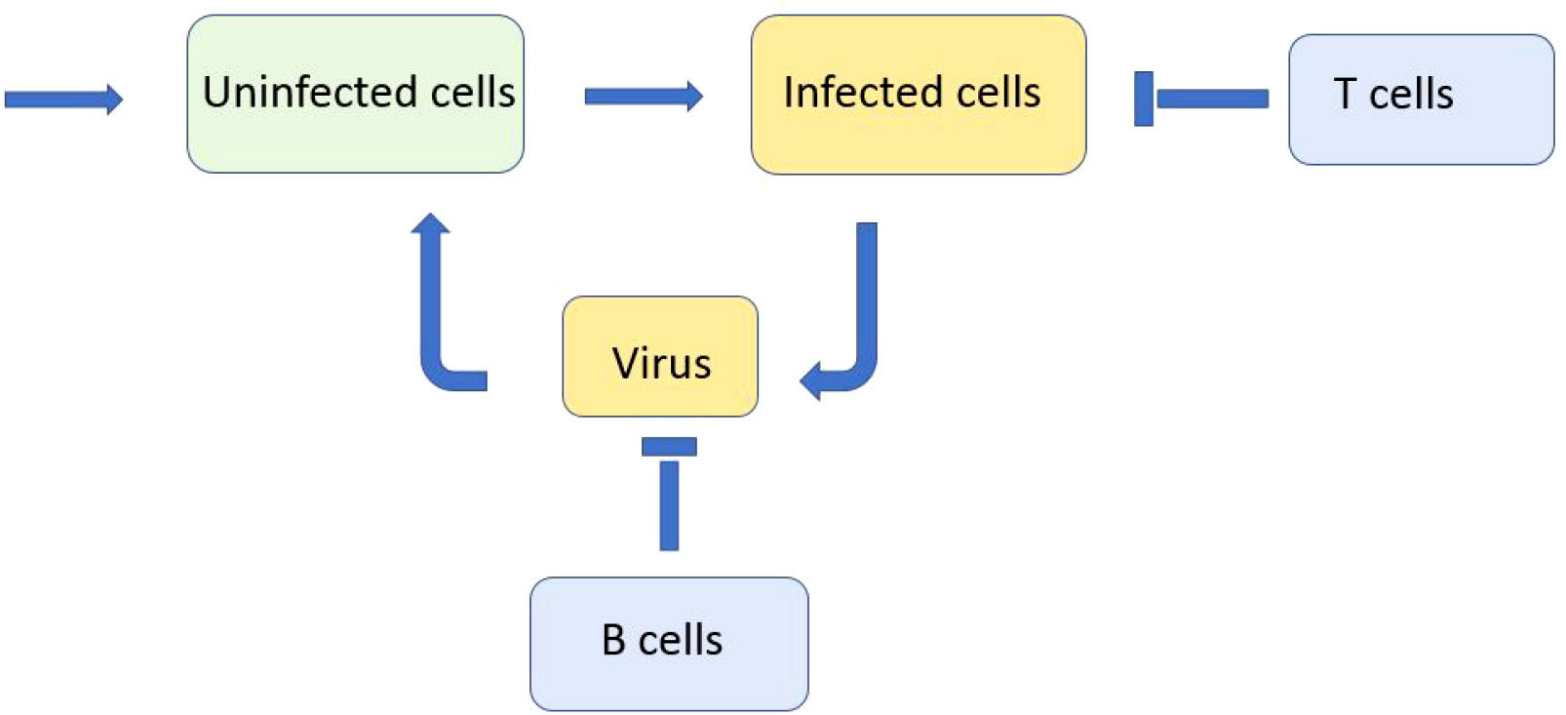
Schematic representation of a simplified model of infection progression and immune response. Virus replicates in infected cells and infects uninfected cells. Infected cells are eliminated by cytotoxic T lymphocytes and virus is neutralised by antibodies produced by B lymphocytes.


We consider a reaction–diffusion system of equations for the concentrations of uninfected cells, infected cells, viruses, and immune cells. We deliberately consider a minimal model in order to reveal the main qualitative effects without hiding them by secondary details (**Figure 1**). We will discuss the model assumptions and simplifications below. We begin with the model of virus evolution in the space of genotypes that will be used to describe the emergence of new variants (strains and quasi-species). In this case, the space variable *x* corresponds to a virus genotype. The concentration of uninfected cells *U*(*t*) does not depend on the space variable; the concentration of infected cells *W*(*x,t*) depends on it. Equation (2.1) for uninfected cells *U*(*t*)

(2.1) $$\frac{dU}{dt}=\kappa -aUI\left(V\right)-\sigma_{1}U$$

contains the term of their constant production (influx), followed by the terms describing their infection and death. The infection rate of uninfected cells is proportional to the total virus concentration $I\left(V\right)\left(t\right)=\int_{-\infty }^{\infty }V\left(x,t\right)dx$ . We consider here the whole real axis for the mathematical convenience.

Uninfected cells *U*(*t*) infected by virus *V*(*x,t*) become infected *W*(*x,t*) with the same antigenic characterization *x*:


(2.2) $$\frac{\partial W}{\partial t}=aUV-\left(\sigma_{2}^{0}+\sigma_{2}^{1}J\left(C\right)\right)W.$$

The death of infected cells can occur due to their damage by virus replication or due to the immune response with the rate proportional to the integral $J\left(C\right)\left(t\right)=\int_{-\infty }^{\infty }\phi \left(x-y\right)C\left(y,t\right)dy$ . Immune cells *C*(*x*,*t*) have the same antigenic characterization *x* as virus *V*(*x*,*t*) , and the kernel *ϕ*(*x*−*y*) characterizes the cross-reactivity of the immune response, that is, the efficacy of immune cells *C*(*y*,*t*) in the elimination of infected cells *W*(*x*,*t*) .

Virus concentration *V*(*x*,*t*) depends on its antigenic characterization *x*. The first term in the right-hand side of the equation


(2.3) $$\frac{\partial V}{\partial t}=D\frac{\partial^{2}V}{\partial x^{2}}+bW-\left(\sigma_{3}^{0}+\sigma_{3}^{1}J\left(C\right)\right)V$$

describes its mutation, that is, diffusion in the space of genotypes, the second term corresponds to its replication in infected cells, and the last term represents its natural death and its neutralization by antibodies. For the simplicity of presentation, we do not introduce an additional equation for the antibody concentration and suppose that it is proportional to the concentration of the cells of the adaptive immune response. As before, we take into account the cross-reaction of the immune response with the same (or different) kernel.

The cells of the adaptive immune response *C*(*x*,*t*) depend on the antigenic characterization *x*:


(2.4) $$\frac{\partial C}{\partial t}=kV_{\tau }-\sigma_{4}C.$$

We suppose that the rate of their production is proportional to the virus concentration with time delay related to the clonal expansion of immune cells, *V*_*τ*_(*x*,*t*)=*V*(*x*,*t*−*τ*), with the proportionality coefficient *k*=*k*_0_ *C*_0_ including the concentration of naive cells *C*_0_ supposed for simplicity to be constant. The second term in the right-hand side of this equation describes the rate of cell death. In what follows, we will consider this model without time delay (τ = 0) leaving the case with time delay for further studies.

The system of equations (2.1)–(2.4) represents a simplified but biologically realistic model that will allow us to study the immune escape and emergence of new virus variants (Section 4). We will discuss model simplifications and limitations in Section 6. When a new variant appears, it starts to spread in the tissue in the competition with other variants. We will study these processes in Section 5 with a similar but modified model.

## 3 Basic properties of the model

In this section, we will establish some basic properties of the model, such as its stationary points and their stability for the Ordinary Differential Equations (ODE) system or wave propagation for the spatial system. They give a general understanding of infection progression and will be used below as a basis for a more detailed analysis. We begin the analysis of viral infection progression with the basic model


(3.1) $$\frac{dU}{dt}=\kappa -aUV-\sigma_{1}U,$$

(3.2) $$\frac{dW}{dt}=aUV-\sigma_{2}W,$$

(3.3) $$\frac{dV}{dt}=bW-\sigma_{3}V$$

for the concentrations of uninfected cells *U*, infected cells *W*, and virus *V*. The right-hand side of equation (3.1) describes the constant influx of cells, their infection by a virus, and their death. In the absence of a virus, this equation provides a constant concentration of uninfected cells in the tissue.

### 3.1 Infection progression in cell culture

The average life span of epithelial cells is estimated up to several months (39) being much longer than the multiplicity of virus assays. Taking into account that cell division is suppressed in the culture, we can neglect cell influx and death in the model. Setting  *κ*=*σ*_1_=0 , we divide equation (3.1) by *U* and integrate from 0 to infinity:


(3.4) $$\ln\;\left(\frac{U_{f}}{U_{0}}\right)=-a\int_{0}^{\infty }V\left(t\right)dt.$$

Here, *U*_0_=*U*(0),*U*_*f*_=*U*(*∞*) . Next, assuming that *W*(0)=*W*(*∞*)=0 , we take a sum of equations (3.1) and (3.2) and integrate


(3.5) $$U_{f}-U_{0}=-\sigma_{2}\int_{0}^{\infty }W\left(t\right)dt.$$

Finally, assuming that *V*(0)=*V*(*∞*)=0 , and integrating equation (3.3), we obtain


(3.6) $$b\int_{0}^{\infty }W\left(t\right)dt=\sigma_{3}\int_{0}^{\infty }V\left(t\right)dt.$$

We obtain from equations (3.4)-(3.6) the equation with respect to *ω*=*U*_*f*_/*U*_0_ :


(3.7) $$\ln\;\omega =R_{0}\left(\omega -1\right),$$

where *R*_0_=*abU*_0_/(*σ*_2_ *σ*_3_) is the virus replication number. This equation has a solution *ω*∈(0,1) if and only if *R*_0_>1 . If *R*_0_<1 , then, the only solution of this equation is *ω*=1 . In this case, *U*_*f*_=*U*_0_ , which means that infection does not develop. Let us note that the virus replication number is similar here to the basic reproduction number in the epidemiological model Susceptible, Exposed, Infected, Recovered (SEIR), and equation (3.7) is also the same.

We can now determine the total viral load $V_{T}=\int_{0}^{\infty }V\left(t\right)dt$ from equation (3.4). In order to obtain a more explicit expression, let us note that for the values of *R*_0_ that are large enough, the solution *ω* of equation (3.7) is small, *ω*≪1 . Then, ln *ω* approximately equals –*R*_0_, and *V*_*T*_=*R*_0_/*a* . The total viral load characterizes virus infectivity, that is, the rate of infection transmission from infected to uninfected individuals (31).

### 3.2 Infection progression in tissue

Let us now consider system (3.1)–(3.3) with the turnover of uninfected cells, *κ*,*σ*_1_≠0 . It has an infection-free equilibrium point *U*_*n*_=*κ*/*σ*_1_,*W*=*V*=0 and an endemic equilibrium point


(3.8) $$U_{e}=\frac{\sigma_{2}\sigma_{3}}{ab},\;V_{e}=\left(\frac{ab\kappa }{\sigma_{1}\sigma_{2}\sigma_{3}}-1\right)\frac{\sigma_{1}}{a},\;W_{e}=\left(\frac{ab\kappa }{\sigma_{1}\sigma_{2}\sigma_{3}}-1\right)\frac{\sigma_{1}\sigma_{3}}{ab}.$$

Along with virus replication number *R*_0_ for cell culture, we now introduce its analog for living tissue


$$R_{v}=U_{n}/U_{e}=\frac{ab\kappa }{\sigma_{1}\sigma_{2}\sigma_{3}}.$$

**Figure 2:**
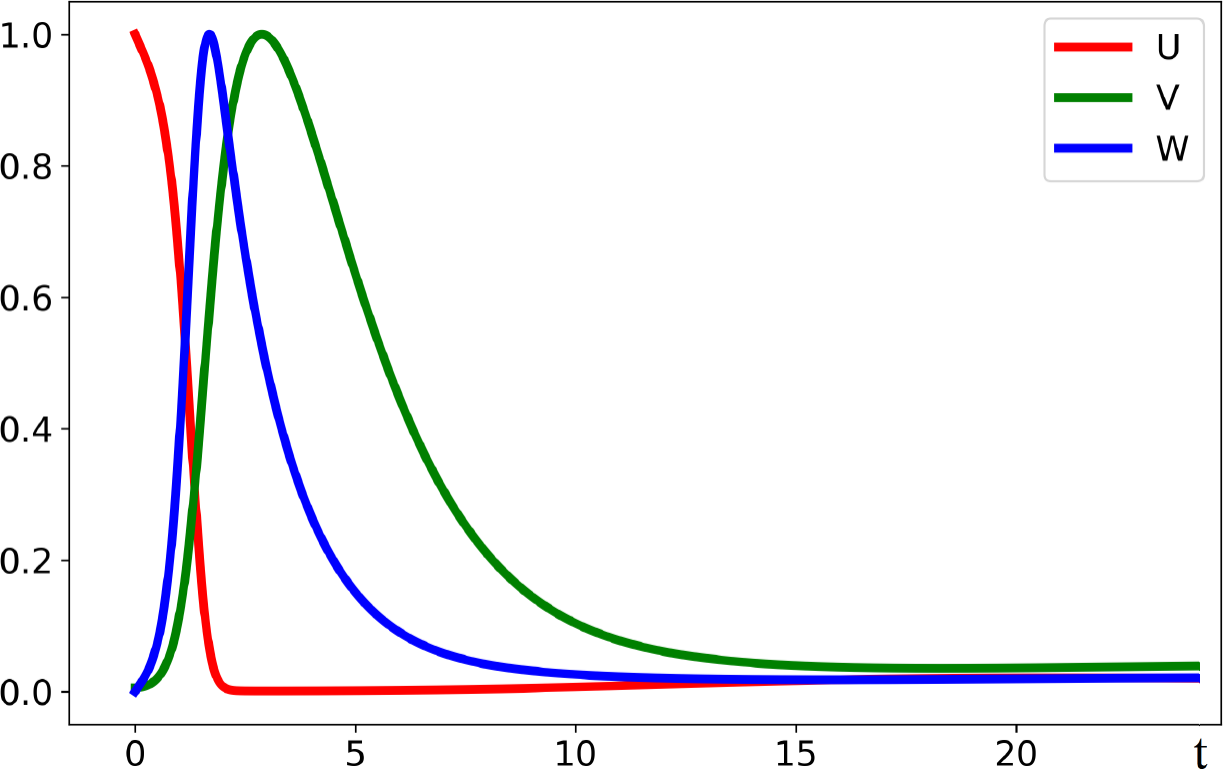
Dynamics of solutions described by system (3.1)–(3.3). If the virus replication number *R_v_* is larger than 1, the concentration of uninfected cells *U*(*t*) decreases, while the concentrations of infected cells *W*(*t*) and of virus *V*(*t*) first increase and later decrease converging to the stationary point. All curves are normalized to their maximum. The values of parameters are as follows: *a* = 0.2, *b* = 100, *κ* = 0.01, *σ*_1_ = 0.1, *σ*_2_ = 0.65, and *σ*_3_ = 0.6.

Hence, the endemic equilibrium is positive if and only if *R*_*v*_>1 . Furthermore, the same condition provides the instability of the normal equilibrium point and infection progression (**Figure 2**). It starts with virus outbreak (acute stage) and then converges to the endemic equilibrium (chronic stage). We do not take into account here the influence of the adaptive immune response on infection progression, which can eliminate it after the acute stage.

### 3.3 Antigen-dependent infection progression

Taking into account virus density distribution in the genotype space, we introduce the space variable *x* and consider the system of equations


(3.9) $$\frac{dU}{dt}=\kappa -aUI\left(V\right)-\sigma_{1}U,$$

(3.10) $$\frac{\partial W}{\partial t}=aUV-\sigma_{2}W,$$

(3.11) $$\frac{\partial V}{\partial t}=D\frac{\partial^{2}V}{\partial x^{2}}+bW-\sigma_{3}V,$$

where *U*(*t*) depends only on time and *V*(*x*,*t*) and *W*(*x*,*t*) depend also on the space variable *x*. If we consider this system of equations on a bounded space interval with no-flux boundary conditions for *V*, that is, $\frac{\partial V}{\partial x}=0$ at the boundaries of the interval, then we can integrate it with respect to *x* and reduce to the previous space-independent model.

### 3.4 Adaptive immune response

We consider the previous model (3.1)–(3.3) completed by a simplified model of the adaptive immune response:


(3.12) $$\frac{dU}{dt}=\kappa -aUV-\sigma_{1}U,$$

(3.13) $$\frac{dW}{dt}=aUV-\left(\sigma_{2}^{0}+\sigma_{2}^{1}C\right)W,$$

(3.14) $$\frac{dV}{dt}=bW-\left(\sigma_{3}^{0}+\sigma_{3}^{1}C\right)V,$$

(3.15) $$\frac{\partial C}{\partial t}=kV_{\tau }-\sigma_{4}C.$$

Here *C* is the concentration of immune cells. In order to keep the model sufficiently simple, we consider here only one type of immune cells and assume that they act on infected cells as cytotoxic T lymphocytes in equation (3.13) and on the virus through B cells and neutralizing antibodies in equation (3.14).

In order to determine the stationary points of system (3.12)–(3.15), we express *C*,*W*, and *U* through *V*:


$$C=\frac{K}{\sigma_{4}}V,\;W=\frac{1}{b}\left(\sigma_{3}^{0}+\sigma_{3}^{1}\frac{k}{\sigma_{4}}V\right)V,U=\frac{\kappa }{aV+\sigma_{1}}.$$

Hence, there is an infection-free stationary point *U*_*n*_=*κ*/*σ*_1_,*W*=*V*=*C*=0 and an endemic stationary point for which *V*≠0 , and it can be found from the equation


(3.16) $$\left(1+\frac{a}{\sigma_{1}}V\right)\left(1+\frac{\sigma_{2}^{1}k}{\sigma_{2}^{0}\sigma_{4}}V\right)\left(1+\frac{\sigma_{3}^{1}k}{\sigma_{3}^{0}\sigma_{4}}V\right)=\frac{ab\kappa }{\sigma_{1}\sigma_{2}^{0}\sigma_{3}^{0}}.$$

If the virus replication number $R_{v}=\frac{ab\kappa }{\sigma_{1}\sigma_{2}^{0}\sigma_{3}^{0}}$ is larger than 1, then this equation has a single positive solution. Let us note that *R_v_* does not depend on $\sigma_{2}^{1}$ and $\sigma_{3}^{1}$ , that is, on infection elimination by the adaptive immune response. However, the solution of equation (3.16) depends on these parameters. As it can be expected, it decreases with the increase of infection elimination.

## 4 Immune escape and emergence of new virus variants

In order to simplify the analysis of the model and the interpretation of the results, we neglect the depletion of host cells and replace *U* in equation (2.2) by *U*_0_.

### 4.1 B cells in the immune response

Let us first consider the case where the elimination of infected cells by the immune cells is neglected, $\sigma_{2}^{1}=0$ , and the immune response acts only through B cells and neutralizing antibodies in the equation for the virus concentration. We obtain the following system of equations:


(4.1) $$\frac{\partial W}{\partial t}=aU_{0}V-\sigma_{2}W,$$

(4.2) $$\frac{\partial V}{\partial t}=D_{1}\frac{\partial^{2}V}{\partial x^{2}}+bW-\left(\sigma_{3}^{0}+\sigma_{3}^{1}J\left(C\right)\right)V,$$

(4.3) $$\frac{\partial C}{\partial t}=kV-\sigma_{4}C,$$

where the superscript in $\sigma_{2}^{0}$ is omitted for the simplicity of notation.

This system has a normal equilibrium *W*=*V*=*C*=0 (no infection) and an endemic equilibrium


$$W_{0}=\frac{aU_{0}}{\sigma_{2}}V_{0},\;V_{0}=\left(R_{v}-1\right)\;\frac{\sigma_{3}^{0}\sigma_{4}}{k\sigma_{3}^{1}},\;C_{0}=\frac{k}{\sigma_{4}}V_{0},$$

where $R_{v}=abU_{0}/\left(\sigma_{2}\sigma_{3}^{0}\right)$ is the virus replication number. If *R*_0_>1, then the endemic equilibrium is positive.

**Figure 3:**
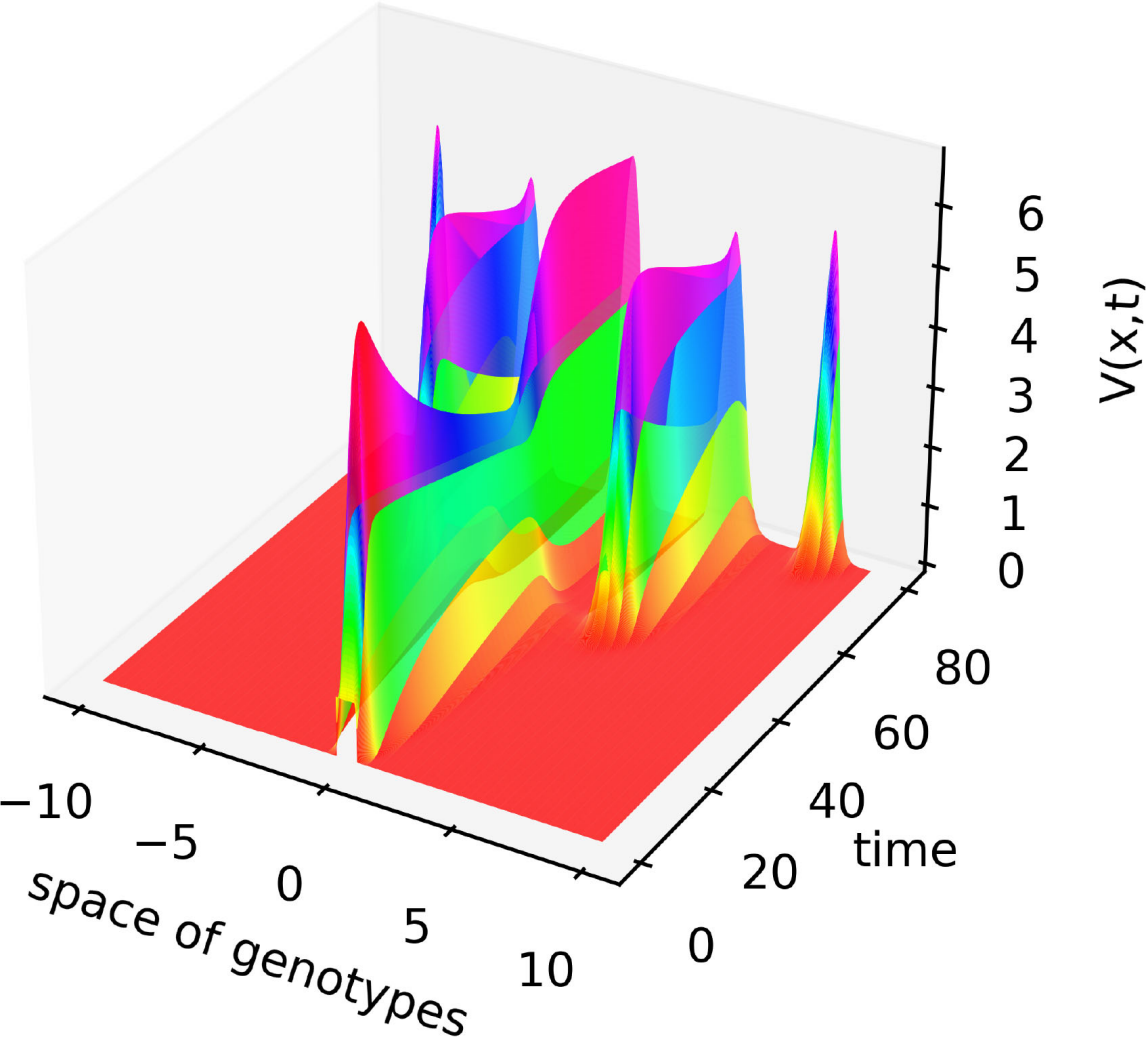
Solution *V*(*x*,*t*) of system (4.1)–(4.3) illustrating the emergence of new virus variants for the values of parameters are as follows: L=10 , D=0.005 , k=0.5 , a=1 , $b=2,\sigma_{2}^{0}=50.0$ , $\sigma_{2}^{1}=80$ , $\sigma_{3}^{0}=1$ , $\sigma_{3}^{1}=8$ , $\sigma_{4}=4$ , the half-support of the kernel (4.10) *N*=1.

**Figure 4:**
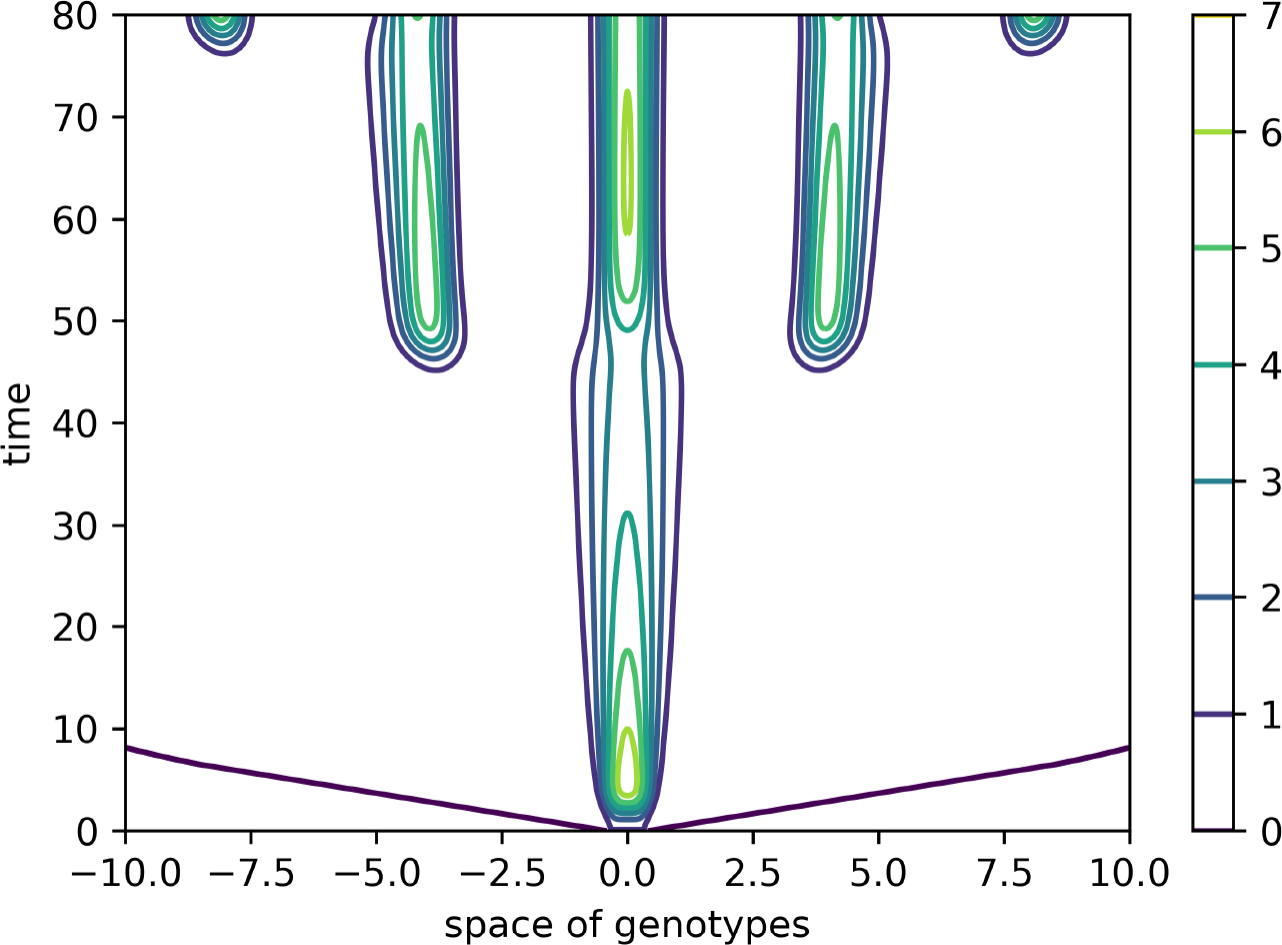
Level lines of the function *V*(*x*,*t*) in **Figure 3** on the (*x*,*t*)-plane. The support of the initial condition is located at the center of the interval. After some time, new peaks of the virus density distribution emerge from both sides of it. The same values of parameters as in **Figure 3**.


The linear stability analysis of system (4.1)–(4.3) shows that this stationary point can become unstable leading to the emergence of spatial structures (**Appendix 1**). An example of the numerical simulations of system (4.1)–(4.3) is shown in **Figures 3**, **4**. We consider a piecewise constant kernel of the integral *J(C)*:

(4.4) $$\phi \left(x\right)=\frac{1}{2N}\{\begin{array}{ccc} 1 & , & \left|x\right|\leq N\\ 0 & , & \left|x\right|>N \end{array}.$$

In this case, the Fourier transform $\tilde{\phi }\left(\xi \right)=\sin\;(N\xi )/\left(N\xi \right)$ has an alternating sign. Therefore, instability occurs for the sufficiently small mutation rate *D* leading to the emergence of localized peaks of solution. If the initial condition is given by a localized function with a narrow support at the center of the interval, then, new peaks of the solution emerge after some time from both sides of the initial distribution. From the mathematical point of view, this dynamic corresponds to the propagation of a periodic wave. Biologically, they correspond to the emergence of new virus variants (strains and quasi-species) that represent virus density distribution in the genotype space localized around different genotypes.

Let us note that numerical simulations are carried out on a bounded interval. In order to eliminate the influence of the boundary, periodic boundary conditions are considered. The space integrals are taken over the bounded interval. One more remark concerns the reduction of system (4.1)–(4.3) to a single equation previously studied in (24). Such reduction can be done if we use quasi-stationary approximations for the concentrations of infected cells and immune cells in equations (4.1) and (4.3). Then, we express *W* and *C* through *V* and substitute in equation (4.2).

### 4.2 T and B cells

Consider now the case where the adaptive immune response also acts on the elimination of infected cells through cytotoxic T lymphocytes:


(4.5) $$\frac{\partial W}{\partial t}=aU_{0}V-\left(\sigma_{2}^{0}+\sigma_{2}^{1}J_{1}\left(C\right)\right)W,$$

(4.6) $$\frac{\partial V}{\partial t}=D\frac{\partial^{2}V}{\partial x^{2}}+bW-\left(\sigma_{3}^{0}+\sigma_{3}^{1}J_{2}\left(C\right)\right)V,$$

(4.7) $$\frac{\partial C}{\partial t}=kV-\sigma_{4}C.$$

Here, the kernels *ϕ*_*i*_(*x*) of the integrals $J_{i}\left(C\right)=\int_{-\infty }^{\infty }\phi_{i}\left(x-y\right)C\left(y,t\right)dy$ can be different. The integrals are taken on a bounded interval in numerical simulations.

**Figure 5:**
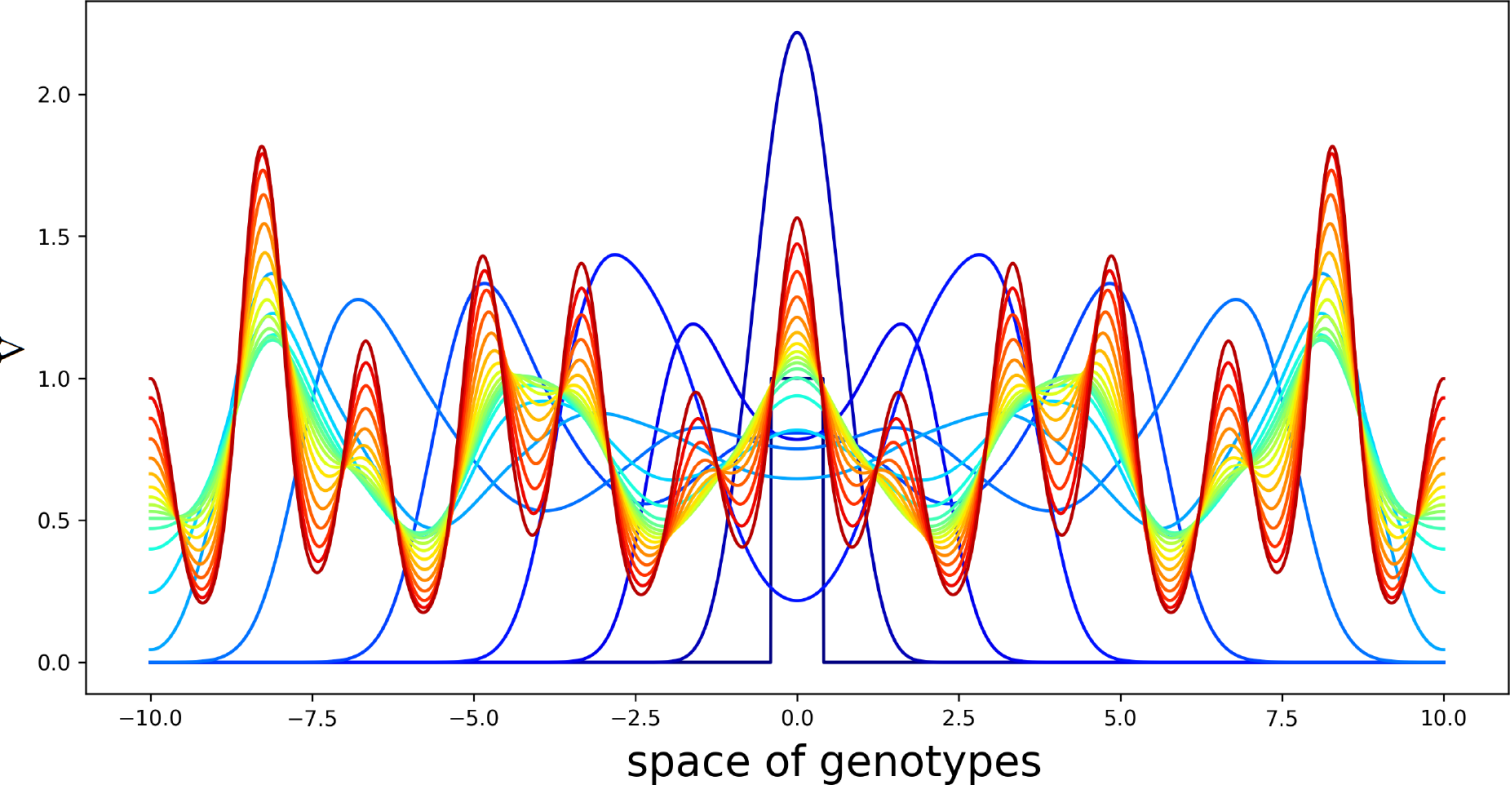
Solution *V*(*x*,*t*) of system (4.5)–(4.7) in consecutive moments of time. Different colors from blue to red correspond to increasing time. Initial condition is a piecewise constant function with the support at the center of the interval. The values of the parameters are as follows: L=10 , D=0.005 , k=0.5 , a=1 , b=2 , $\sigma_{2}^{0}=50.0$ , $\sigma_{2}^{1}=80$ , $\sigma_{3}^{0}=1$ , $\sigma_{3}^{1}=8$ , $\sigma_{4}=4$ . The half-supports of the kernels are *N*=1 and *N*=3 .

The linear stability analysis of this problem is similar to the previous one. We do not present it here for brevity. An example of numerical simulations for two different kernels [both similar to (4.10)] is shown in **Figure 5**. The initial condition in this simulation is a piecewise constant function *V*(*x*,0) with the support at the center of the interval, *U*(*x*,0)=*U*_0_ , *W*(*x*,0)=0 .

Virus concentration spreads from the center of the interval in both directions as a periodic wave converging to a stationary periodic in space solution behind the wave. Since the kernels of the integrals *J*_1_(*C*) and *J*_2_(*C*) are different from each other, this structure has a double periodicity. The values of parameters are chosen here for the illustration of instability and pattern formation.

## 5 Infection spreading in epithelial tissue

### 5.1 Two-virus model

**Figure 6:**
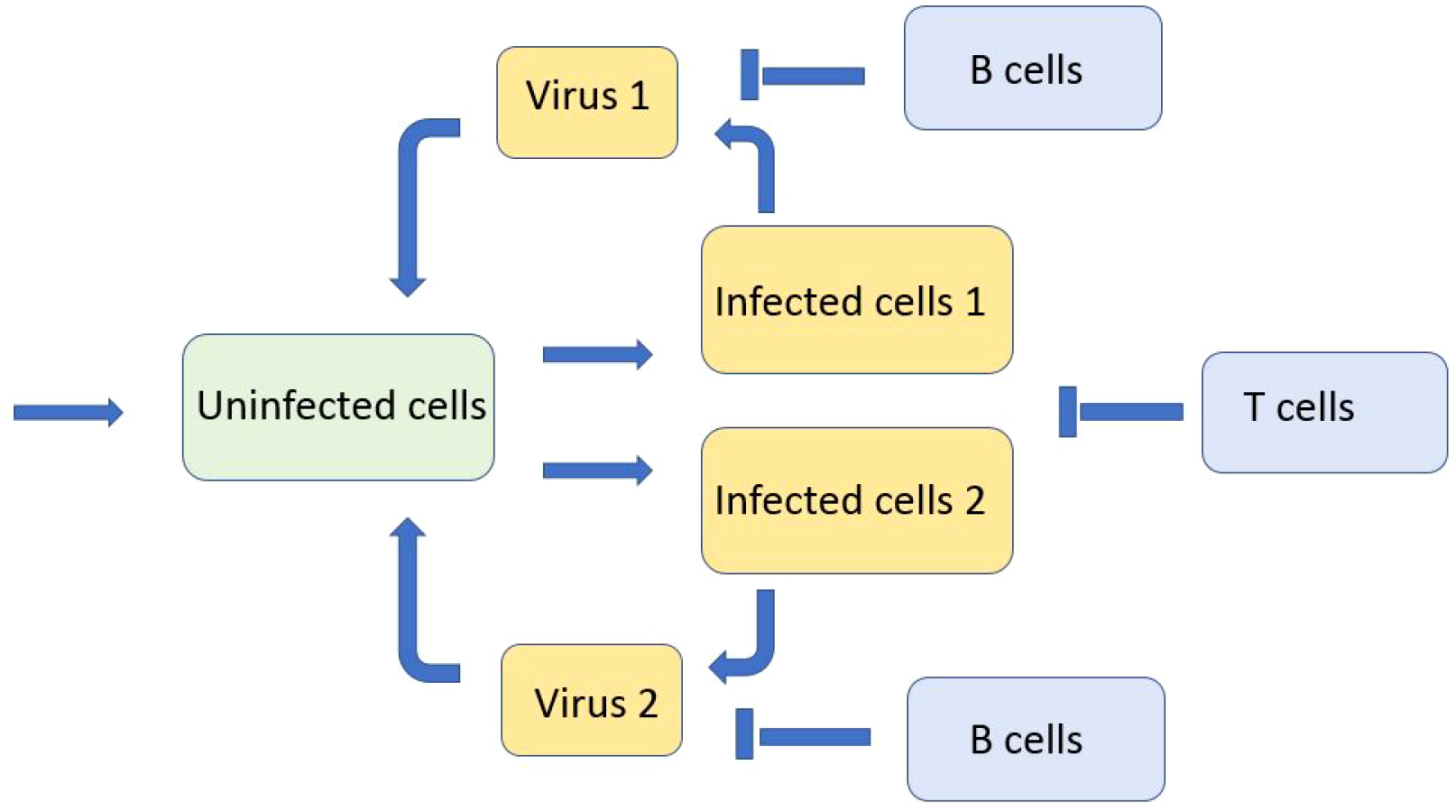
Schematic representation of the model with two viruses competing for uninfected cells. There are two types of infected cells corresponding to the virus type. Both types of viruses and infected cells are eliminated by the immune response.


Emerging virus variants are characterized by a relatively narrow distribution in the genotype space. We will now consider a discrete genotype space with two virus variants in order to study their competition during infection progression in the infected tissue. In this case, the space variable *x* corresponds to the spatial coordinate in the tissue and not to the virus genotype considered above. We consider the system of equations for the concentrations of uninfected cells *U*(*x*,*t*) , two types of viruses (variants) *V*_1_(*x*,*t*) and *V*_2_(*x*,*t*) , two types of infected cells *W*_1_(*x*,*t*) and *W*_2_(*x*,*t*) corresponding to these viruses, and the concentration of immune cells *C*(*t*) (**Figure 6**):

(5.1) $$\frac{\partial U}{\partial t}=\kappa -a_{1}UV_{1}-a_{2}UV_{2}-\sigma_{1}U,$$

(5.2) $$\frac{\partial W_{1}}{\partial t}=a_{1}UV_{1}-\left(\sigma_{21}^{0}+\sigma_{21}^{1}C\right)W_{1},$$

(5.3) $$\frac{\partial W_{2}}{\partial t}=a_{2}UV_{2}-\left(\sigma_{22}^{0}+\sigma_{22}^{1}C\right)W_{2},$$

(5.4) $$\frac{\partial V_{1}}{\partial t}=D\frac{\partial^{2}V_{1}}{\partial x^{2}}+b_{1}W_{1}-\left(\sigma_{31}^{0}+\sigma_{31}^{1}C\right)V_{1},$$

(5.5) $$\frac{\partial V_{2}}{\partial t}=D\frac{\partial^{2}V_{2}}{\partial x^{2}}+b_{2}W_{2}-\left(\sigma_{32}^{0}+\sigma_{32}^{1}C\right)V_{2},$$

(5.6) $$\frac{dC}{dt}=k_{1}I\left(V_{1}\right)+k_{2}I\left(V_{2}\right)-\sigma_{4}C.$$

In the analysis, this system is considered on the whole real axis, while in numerical simulations, on a bounded interval with the no-flux boundary conditions for the virus concentrations. The integrals $I\left(V_{i}\right)=\int_{-\infty }^{\infty }V_{i}\left(x,t\right)dx$ are taken over the whole axis in the first case and over the bounded interval in the second case. We suppose that the production of immune cells occurs in other organs (thymus and lymph nodes). It is proportional to the total virus concentrations and does not depend on the space variable. We begin the analysis of this model with a single virus, and we will determine its spreading speed and viral load. Then, we will proceed to the investigation of virus competition.

### 5.2 Spreading speed for a single virus

We determine in this section the infection spreading speed for a single virus *V*_1_. We begin with the case without the immune response, that is, *k*_1_=0 , and, as a consequence, *C*=0 in equations (5.2) and (5.4). Therefore, we consider the following system of equations


(5.7) $$\frac{\partial U}{\partial t}=\kappa -a_{1}UV_{1}-\sigma_{1}U,$$

(5.8) $$\frac{\partial W_{1}}{\partial t}=a_{1}UV_{1}-\sigma_{21}W_{1},$$

(5.9) $$\frac{\partial V_{1}}{\partial t}=D\frac{\partial^{2}V_{1}}{\partial x^{2}}+b_{1}W_{1}-\sigma_{31}V_{1}$$

on the whole axis. For the simplicity of notation, we omit the superscript in the coefficients $\sigma_{21}^{0}$ and $\sigma_{31}^{0}$ . We look for its solution in the form *U*(*x*,*t*)=*u*(*x*−*ct*),*V*_1_(*x*,*t*)=*v*(*x*−*ct*),*W*_1_(*x*,*t*)=*w*(*x*−*ct*), where *c* is the wave speed. We obtain the following problem:


(5.10) $$cu^{\prime }+\kappa -a_{1}uv-\sigma_{1}u=0,$$

(5.11) $$cw^{\prime }+a_{1}uv-\sigma_{21}w=0,$$

(5.12) $$Dv^{\prime \prime }+cv^{\prime }+b_{1}w-\sigma_{31}v=0,$$

(5.13) $$\begin{array}{l} u\left(-\infty \right)=u_{e},\;w\left(-\infty \right)=w_{e},\;v\left(-\infty \right)=v_{e},\\ u\left(\infty \right)=u_{0},\;w\left(\infty \right)=0,\;v\left(\infty \right)=0. \end{array}$$

Here, *u*_0_=*κ*/*σ*_1_ , the values *u*_*e*_,*w*_*e*_,*v*_*e*_ are determined in (3.8), and the prime symbol denotes the derivative with respect to *ξ*=*x*−*ct* .

We will determine the wave speed *c* by the linearization method. We set *u*=*u*_0_=*κ*/*σ*_1_ and look for a solution of equations (5.11) and (5.12) in the form


$$w\left(x\right)=pe^{-\lambda x},\;v\left(x\right)=qe^{-\lambda x},\;\lambda >0.$$

Substituting these expressions into the equations, we obtain


$$-c\lambda p+a_{1}u_{0}q-\sigma_{21}p=0,\;D\lambda^{2}q-c\lambda q+b_{1}p-\sigma_{31}q=0.$$

We exclude *p* and *q* and obtain the equation with respect to *λ*:


$$D\lambda^{2}-c\lambda +\frac{a_{1}b_{1}u_{0}}{c\lambda +\sigma_{21}}-\sigma_{31}=0.$$

We set *μ*=*λc* . Then, from the last equation, we obtain


$$c^{2}=\frac{D\mu^{2}\left(\mu +\sigma_{21}\right)}{\left(\mu +\sigma_{31}\right)\left(\mu +\sigma_{21}\right)-a_{1}b_{1}u_{0}}.$$

Since *σ*_21_ *σ*_31_<*a*_1_ *b*_1_ *u*_0_ (*R*_*v*_>1), then the denominator of the last expression equals 0 for some *μ*=*μ*_0_>0 , and it is positive for *μ*>*μ*_0_. The minimal wave speed *c*_0_ is given by the expression


(5.14) $$c_{0}=\underset{\mu >\mu_{0}}{\min}\sqrt{\frac{D\mu^{2}\left(\mu +\sigma_{21}\right)}{\left(\mu +\sigma_{31}\right)\left(\mu +\sigma_{21}\right)-a_{1}b_{1}u_{0}}}.$$

Let us note that the minimal wave speed depends on parameters *κ* and *σ*_1_ through their ratio *u*_0_=*κ*/*σ*_1_ .

In order to take into account the dependence of the wave speed on the immune response, we express *C*=*k*_1_ *I*(*V*_1_)/*σ*_4_ from equation (5.6) (for the wave propagation). Then


(5.15) $$\sigma_{21}=\sigma_{21}^{0}+\sigma_{21}^{1}C=\sigma_{21}^{0}+\frac{\sigma_{21}^{1}k_{1}}{\sigma_{4}}I\left(V_{1}\right),\sigma_{31}=\sigma_{31}^{0}+\sigma_{31}^{1}C=\sigma_{31}^{0}+\frac{\sigma_{31}^{1}k_{1}}{\sigma_{4}}I\left(V_{1}\right).$$

Hence, the coefficients *σ*_21_ and *σ*_31_ in (5.14) depend on the viral load *I*(*V*_1_) . We determine it in the next section.

### 5.3 Viral load for a single virus

In order to determine analytically viral load *I*(*V*_1_) in problem (5.10)–(5.13), we set *κ*=0 , *σ*_1_=0 . Let us note that the viral load weakly depends on these coefficients if they are small enough, and the wave speed does not depend on their variation if their ratio remains constant. In the considered case, *v*_*e*_=*w*_*e*_=0 in (5.13).

Dividing equation (5.10) and integrating over the whole axis, we obtain


(5.16) $$c\ln\;\frac{u_{0}}{u_{e}}=aI\left(v\right).$$

Next, taking the sum of equations (5.10) and (5.11) and integrating, we get


(5.17) $$c\left(u_{0}-u_{e}\right)=\sigma_{21}I\left(w\right).$$

Finally, from equation (5.12),


(5.18) $$bI\left(w\right)=\sigma_{31}I\left(v\right).$$

The system of equations (5.16)-(5.18) contains three unknowns: *u*_*e*_, *I*(*v*), and *I*(*w*). We can reduce it to a single equation with respect to the variable *z*=*I*(*v*) :


(5.19) $$bcu_{0}\left(1-e^{-az/c}\right)=z\left(\sigma_{21}^{0}+\frac{k_{1}\sigma_{21}^{1}}{\sigma_{4}}z\right)\left(\sigma_{31}^{0}+\frac{k_{1}\sigma_{31}^{1}}{\sigma_{4}}z\right),$$

where we use (5.15).

This equation has solution *z*=0 for all values of parameters. Furthermore, it has a positive solution if the derivative of its left-hand side at *z*=0 exceeds the derivative of the right-hand side. This condition has the form *R*_*v*_>1 . Let us note that the virus replication number *R_v_* and the condition of the existence of a positive solution of equation (5.19) do not depend on the parameters of the immune response. However, if this condition is satisfied, then the value of the positive solution depends on the immune response. This means that the immune response does not stop infection from spreading (if *σ*_4_>0), but it decreases the viral load.

Consider first the case without the immune response for which *k*_1_=0 . Since *z*=*I*(*v*) is sufficiently large, such that *az*/*c*≫1 , then we obtain from equation (5.19):


(5.20) $$I\left(v\right)\approx \frac{bcu_{0}}{\sigma_{21}^{0}\sigma_{31}^{0}}=cR_{v}/a.$$

This analytical formula is compared with numerical simulations in **Table 1**. If *k*_1_≠0 , then we need to solve the system of equations (5.14) and (5.19) with respect to *c*=*c*_0_ and *z*=*I*(*v*). Since it does not admit a simple analytical solution, we use a combination of analytical and numerical results. Namely, we solve equation (5.19) with respect to the viral load substituting in this equation the wave speed determined in direct numerical simulations of system (5.1)–(5.6) (for a single virus). The analytical and numerical results are in good agreement with a slight difference between them due to numerical accuracy.

**Table 1:** Analytical and numerical values of the viral load.

*log* *b*	*k*_1_=0 (analyt/numer)	*k*_1_=0.1 (analyt/numer)	*k*_1_=1 (analyt/numer)
3	1540/1540	20.42/20.46	4.36/4.38
3.5	6641/6686	35.12/35.30	7.43/7.45
4	28300/28700	59.49/59.63	12.23/12.45

For *k*_1_=0, the analytical value is given by formula (5.20) where the wave speed is determined by formula (5.14). For *k*_1_=0.1 and *k*_1_=1, the analytical value is found from formula (5.19) where the wave speed is taken from the numerical simulations. The numerical values of viral load are found by the direct numerical simulations of system (5.1)–(5.6) with the values of parameters $a_{1}=0.1,\kappa =0,D=0.001,\sigma_{1}=0,\sigma_{21}^{0}=\sigma_{21}^{1}=\sigma_{31}^{1}=0.1,\sigma_{31}^{0}=1,\sigma_{4}=0.1$ .

**Figure 7:**
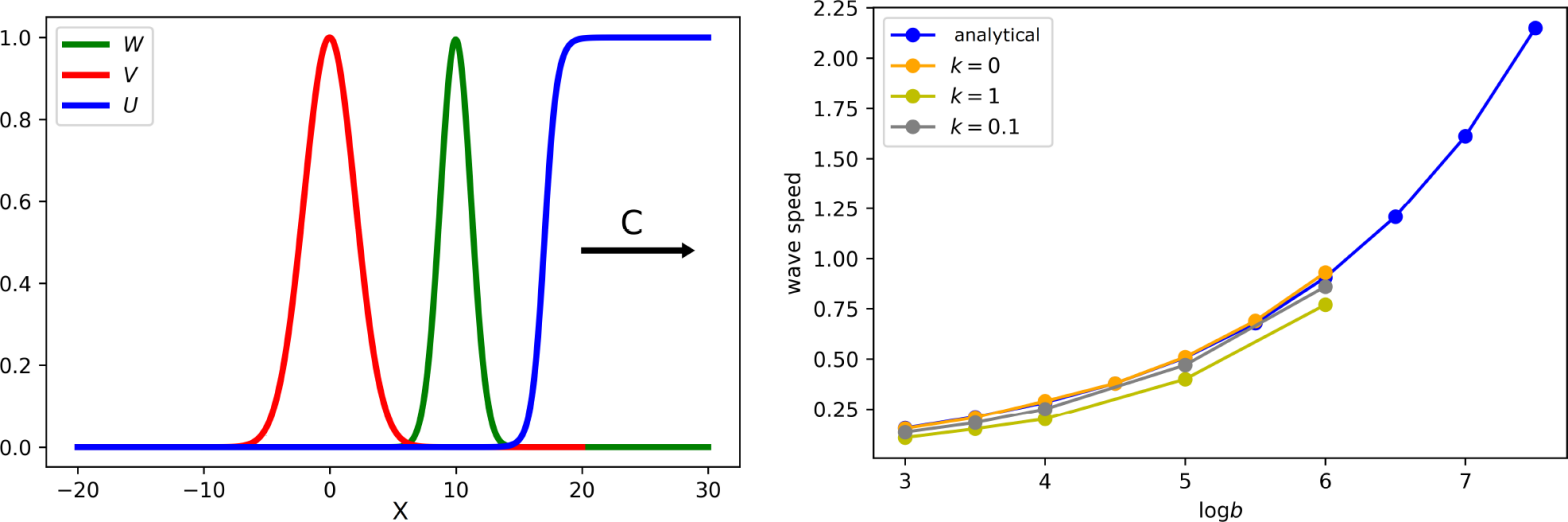
Left: snapshot of the solution of system (5.7)–(5.9). If the virus replication number *R_v_* is larger than 1, infection spreads as a reaction–diffusion wave with speed *c*. All curves are normalized to their maximum. Right: wave speed as a function of virus replication rate *b* for different rates of the clonal expansion of immune cells. Analytical value for *k*_1_ = 0 is obtained by formula (5.14). Numerical values are found from the solution of system (5.1)–(5.6) (for a single virus) with the values of *k*_1_ indicated in the figure. The values of parameters: $a_{1}=0.1$, κ=0, D=0.001, $\sigma_{1}=0$, $\sigma_{21}^{0}=\sigma_{21}^{1}=\sigma_{31}^{1}=0.1$, ${\text{σ}}_{31}^{0}=1$, $\sigma_{4}=0.1$ .

Comparison of the results for different values of parameter *k*_1_ shows that the immune response weakly influences the spreading speed (**Figure 7**, right), but it has a large influence on the viral load (**Table 1**).

### 5.4 Competition of two variants

Depending on the initial condition, system (5.1)–(5.6) has a solution with a single virus or both of them. In the latter case, they compete for uninfected cells. Numerical simulations show that one of them becomes dominant and eliminates another one due to this competition.

#### 5.4.1 Virus competition without immune response

If we do not take into account the immune response, then the dominance condition is determined by the individual spreading speed. We can formulate this result as follows.

*In the case without immune response (k*_1_=*k*_2_=0*), the variant with a larger individual spreading speed is dominant and eliminates another one. If the spreading speeds are equal to each other, then the two variants coexist.*

Let us note that in the case without the immune response, the individual spreading speed is given by formula (5.14). Hence, we obtain an explicit condition on virus dominance. In particular, for equal death rates, $\sigma_{21}^{0}=\sigma_{22}^{0}$ , $\sigma_{31}^{0}=\sigma_{32}^{0}$ , virus dominance is determined by the product *a*_*i*_ *b*_*i*_ characterizing the virus multiplication rate. If *a*_1_ *b*_1_>*a*_2_ *b*_2_ , then the first variant wins the competition and eliminates the second one. Even a small difference in the values *a*_*i*_ *b*_*i*_ leads to the elimination of one of the variants. However, if they are sufficiently close to each other, then, the disappearance of the “loser” is slow.

Previously, this result was obtained in the case of cell culture (1) where *κ*=*σ*_1_=0 . It remains valid in the case of cell tissue with the positive values of these coefficients. Conditions *k*_1_=*k*_2_=0 mean that immune cells are not produced. A similar result holds for positive *k*_1_ and *k*_2_ and zero death rates $\sigma_{ij}^{1}$ . Proposition formulated above is not proven as a mathematical result because of the limitation of the existing methods of analysis. It is verified in numerical simulations.

#### 5.4.2 Virus competition with immune response

The previous result may not hold in the presence of the immune response. Indeed, let us consider the following example. The first virus variant initiates a weak production of immune cells (*k*_1_=0 in the limiting case), but it is strongly eliminated by the immune response (large values of $\sigma_{21}^{1}$ and $\sigma_{22}^{1}$ ). The second variant has opposite properties: large *k*_2_ and $\sigma_{31}^{1}=\sigma_{32}^{1}=0$ . These conditions mean that the second virus variant initiates a strong immune response that does not act on itself, but it strongly acts on the first variant. For both variants, the individual spreading speed is not influenced by the immune response, and it is given by formula (5.14). The results of the numerical simulations of system (5.1)–(5.6) are presented in **Table 2**. In the case without the immune response, the first variant dominates and eliminates the second one because its individual spreading speed is larger. In the case with the immune response, the second variant dominates because it is less sensitive to the immune response.

**Table 2:** Competition of two virus variants with or without the immune response.

*a*_1_/*a*_2_	speed 1/speed 2	competition *k*_1_=*k*_2_=0	competition *k*_1_=0,*k*_2_=0.1
0.11/0.1	0.158/0.154	*V*_1_ spreads, *V*_2_ stops	*V*_1_ stops, *V*_2_ spreads

The values of the cell infection rates for the two variants are given in the first column and their individual spreading speeds in the second column. Without the immune response, the first variant dominates since its individual speed is larger (third column). With the immune response, the second variant dominates since it is less sensitive to the immune response (fourth column). The values of parameters: $b_{1}=b_{2}=1,000$, κ=0, D=0.001, $\backslash sigma{\_}_{1}=0$, $\sigma_{21}^{0}=\sigma_{31}^{0}=0.1$, $\sigma_{31}^{0}=\sigma_{32}^{0}=1$, $\sigma_{21}^{1}=\sigma_{31}^{1}=0.1$, $\sigma_{22}^{1}$ $=\sigma_{32}^{1}=0,\sigma_{4}=0.1$ .

**Figure 8:**
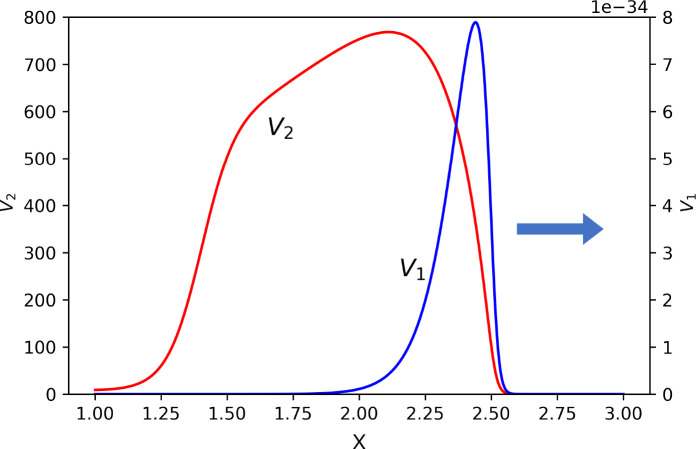
Snapshot of normalized spatial distributions of virus concentrations. The first virus spreads together with the second one, but its maxim concentration and viral load converge to zero. Their respective maximal values are as follows: *V*1 = 10^−31^ and *V*_2_ = 768.5. The values of parameters: *a*1 = 0.15, a_2_ = 0.1, *b*_1_ = *b*_2_ = 1,000, *κ* = 0, *D* = 0.001, *k*_1_ = 0, *k*_2_ = 0.1, *σ*1 = 0, $\sigma_{21}^{0}=\sigma_{31}^{0}=0.1$ , $\sigma_{31}^{0}=\sigma_{32}^{0}=1$ , $\sigma_{21}^{1}=\sigma_{31}^{1}=0.1$ , $\sigma_{21}^{1}=\sigma_{32}^{1}=0$ , *σ*4 = 0.1.

If the dominance of one of the virus variants is sufficiently strong, then another variant completely stops its propagation, and its concentration vanishes. If the dominance is not strong enough, the subdominant variant can also propagate but its concentration and viral load converge to zero (**Figure 8**).

The dynamics of immune cells depend on their production and death rates. If *σ*_4_>0 , then numerical simulations show that *C*(*t*) converges to some limiting value *C*_+_ for a large time. In this case, we can formulate a general result on virus competition in terms of this value. We consider system (5.1)–(5.5) where *C*(*t*) is replaced by *C*_+_ . Then, we can use the previous proposition with modified death rates *σ*_21_ , *σ*_31_ taking into account a constant concentration of immune cells.

*In the case with immune response, consider the death rates (5.15) with the limiting value C*_+_ *of the concentration of immune cells. Then the variant with larger individual spreading speed is dominant and eliminates another one. If the spreading speeds are equal to each other, then the two variants coexist.*

Let us note that the limiting value *C*_+_ cannot be found analytically, although some approximate estimates are possible. If we neglect the death of immune cells (*σ*_4_=0), taking into account the long lifespan of memory cells, then their concentration monotonically grows, even if the virus concentration decreases. In this case, infection spreads with a slowly decaying speed and decreasing viral load.

## 6 Discussion

New virus variants (strains and quasi-species) emerge due to their mutations in the process of virus replication. Constantly appearing new variants compete with each other for uninfected cells (21) and spread in the host organism. We study these processes with mathematical modeling and determine the conditions of the emergence of new virus variants and of their success or failure in the competition.

### 6.1 Emergence of new variants

Virus mutation can give selective advantage increasing virus replication rate or decreasing its death (unrelated to the immune response) leading to the emergence of new viable variants. If the mutations are neutral from the point of view of replication and death rates, they can still be advantageous if they weaken the immune response (infected cell death and virus neutralization) and provide immune escape. We develop in this work a new model of immune escape taking into account virus mutations, considered as diffusion in the space of a genotype, and cross-reactivity in the immune response with its efficacy depending on the genetic distance.

A one-equation model of the emergence of new quasi-species was studied in (22, 24) with a non-local reaction–diffusion equation. This model is qualitatively similar to the model describing the emergence of biological species on the basis of mutations, reproduction, and intraspecific competition (25, 26). In this work, we suggest a more detailed model of the emergence of new variants due to the immune escape. The model takes into account specific features of infection progression with uninfected cells, infected cells, and virus concentration with its replication and death.

A virus exploration of the genetic space can occur in two different modes. The first one can be characterized by a uniform filling of the genetic space without preferential genotypes. From the mathematical point of view, this case corresponds to the propagation of a traveling wave with a stable uniform equilibrium behind the wave. The second mode is characterized by the periodic emergence of new variants characterized by a localized density distribution around some preferential genotypes. This case corresponds to the propagation of periodic waves with a stationary periodic spatial structure behind the wave. In the case of biological species, the second mode gives some evolutionary advantage because the total biomass increases (32). This question is not yet studied for this new model of virus evolution. Another important question concerns the genetic characterization of the emerging variants. In the case of advantageous mutations, new variants emerge in specific locations of the genetic space with the best ratio replication/death. The situation is different for neutral mutations. The location of new peaks of the virus density distribution is determined by the initial condition (initial variant) and the properties of the cross-reactive immune response.

The model of virus evolution suggested in this work can be further developed with a more detailed description of virus replication and of the immune response. Let us note that we studied in this work the emergence of new virus variants taking into account either only B cells in the immune response or the combination of B cells and T cells. The case of T cells only can be considered by the same method. We expect that it can also give the instability and the emergence of structures.

### 6.2 Spreading speed

Viral infection spreads in the infected tissue as a reaction–diffusion wave (see (33) and the references therein). It is characterized by two main parameters: the spreading speed and viral load. There are different methods developed in the theory of reaction–diffusion equations to determine the spreading speed, that is, the speed of reaction-diffusion waves (see (32) and the references therein). In the monostable case, where the wave connects an unstable equilibrium with a stable equilibrium, the traveling wave is not unique. It is shown for various models, including the scalar equation and the monotone systems of equations that waves exist for all values of the speed greater than or equal to some minimal speed *c_0_* (33). The wave with the minimal speed is more interesting for applications because it describes the asymptotic behavior of solutions for a large class of realistic initial conditions (with a finite support).

The minimal speed can be estimated by the linearization method first suggested in (34) for the scalar equation and then used for many different models. The idea of the method is to study the system linearized around the unstable equilibrium and to look for its exponentially decaying positive solution. It appears that such solutions exist for the values of the speed greater than or equal to some speed c_*_ . In general, c_*_≤*c*_0_ , and equality between them or strict inequality depend on a particular problem. In some analytical studies and in numerical simulations, it shown that c_*_=*c*_0_ , that is, the linearization method gives the minimal wave speed (33), although, in some other cases, c_*_ is strictly less than *c*_0_ . In this work, we verify with numerical simulations that these two values coincide: *c*_0_ found numerically converges to the analytical value c_*_ as numerical accuracy is increased.

Thus, the analytical formula allows us to determine the infection spreading speed in the tissue. This result is important by itself and for a further comparison of different virus variants.

### 6.3 Viral load

In the case of a space-independent model, total viral load *V*_*T*_ is understood as the integral of virus concentration over time, $V_{T}=\int_{-0}^{\infty }V\left(t\right)dt$ . In the biological literature, it is related to the area under curve, and it characterizes virus infectivity. In the case of a space-dependent problem, we determine instantaneous viral load *V*_*X*_(*t*) as the integral of virus concentration with respect to the space variable, $V_{X}\left(t\right)=\int_{-\infty }^{\infty }V\left(x,t\right)dx$ . After that, the total viral load can be determined as the time integral of the function *V*_*X*_(*t*) . According to dynamics of the total viral load, infection progression in cell culture has three consecutive stages: decay due to time delay in virus replication, explosive growth when infected cells begin to produce new viral particles, and a constant viral load (or slow growth) during infection spreading (35).

An instantaneous viral load *I*(*v*) during infection spreading can be explicitly determined through the parameters of the model (Section 5.3). It has a particularly simple form in the case without the immune response, $I\left(v\right)=bcu_{0}/\left(\sigma_{21}^{0}\sigma_{31}^{0}\right)$ . This expression depends on the wave speed *c*. The increase of *a* and *b* increases the wave speed and viral load, while the increase of $\sigma_{21}^{0}$ and $\sigma_{31}^{0}$ decreases both of them. However, if we change *a* and *b* in such a way that their product remains constant, then the wave speed *c* in (5.14) does not change, but viral load *I*(*v*) does change. From this point of view, we can state that the spreading speed and viral load are two different and independent characteristics. Although they are expressed through the same model parameters, their values may not correlate, and a high (or low) wave speed can be associated with a high or low viral load. This difference is quite important in the understanding of viral infections, although it is not sufficiently well elucidated in the existing literature.

### 6.4 Symptoms and infectivity

The infection spreading speed determines the part of tissue infected with a virus and, as a consequence, tissue damage. Therefore, spreading speed is correlated with the severity of symptoms. On the other hand, spreading speed in the upper respiratory tract (URT) determines the duration of the incubation period, while the viral load in the URT determines the infection transmission rate in the population (infectivity).

Experimental results on the Delta and Omicron variants of the SARS-CoV-2 infection in the cultures of human epithelial (nasal) and lung cells (4, 5) allow the determination of model parameters (1). After that, modeling can be used to determine the spreading speed and the viral load. Modeling results show that the spreading speed of the Omicron variant is larger than that of the Delta variant in the epithelial cells and smaller in the lung cells. This confirms more severe symptoms of the Delta variant and a smaller incubation period of the Omicron variant. A larger total viral load in the culture of nasal cells corresponds to its higher infectivity.

According to the results presented in this work, the infection spreading speed in tissue is the same as in cell culture, if adaptive immune response is not taken into account. Furthermore, we showed that immune response weakly influences the spreading speed. Therefore, the conclusion about the larger spreading speed of the Omicron variant in the upper respiratory tract and smaller in the lungs, compared to Delta, remains valid. On the other hand, adaptive immune response strongly decreases the value of viral load. However, it becomes fully efficient approximately 6–7 days postinfection. Therefore, it is not so essential from the point of view of infectivity rate.

### 6.5 Virus competition

Once new virus variants emerge, they begin to compete between each other for the host cells. This process can be more efficiently studied if we consider a discrete set of variants and not a continuous genotype variable as before. The main properties of this competition can be elucidated in the case of two variants. It was shown in (1) that virus competition in cell culture is determined by their respective spreading speeds: a virus with a larger individual speed becomes dominant and eliminates another one. This result is in agreement with the experimental data on the competition of Delta and Omicron variants in the cultures of epithelial and lung cells (4).

The dominance condition expressed in terms of the individual spreading speed remains valid in living tissue with the immune response, although the value of the concentration of immune cells cannot be determined analytically. Thus, virus dominance is determined by the individual spreading speed and not by viral load. These are two different infection characterizations that may not be correlated.

Virus dominance is also related to the emergence of new variants. In particular, since Omicron loses the competition with Delta in the culture of lung cells, it is unlikely that it could emerge in the lungs of a chronic patient.

### 6.6 Model limitations and perspectives

We have deliberately considered in this work a simplified model of the immune response without taking into account various cytokines (e.g., interferon and interleukin), cells (e.g., antigen-presenting cells and regulatory T cells), processes (e.g., innate immune response and inflammation), and the involvement of different organs and tissues of the host organism. On the other hand, the model is biologically realistic, and it qualitatively describes the main features of viral infection and immune response. This simplification of the model allows us to obtain sufficiently simple, sometimes analytical results that admit clear biological interpretation. More detailed models can be considered in future investigations. In particular, the combination of adaptive and innate immune responses can provide a better understanding of the dynamics of virus quasi-species.

## Data availability statement

The original contributions presented in the study are included in the article/supplementary material, further inquiries can be directed to the corresponding author.

## Author contributions

VV - concept, analysis, writing. NB - software development. DN - analysis, simulations. VP - analysis, simulations, validation. All authors contributed to the article and approved the submitted version.

## Appendix 1. Linear stability analysis

Linearizing system (4.1)–(4.3) about the point (*W*_0_,*C*_0_,*V*_0_) under the assumption that it is positive, we obtain the linear system

$$\frac{\partial W}{\partial t}=aU_{0}V-\sigma_{2}W,$$

$$\frac{\partial V}{\partial t}=D\frac{\partial^{2}V}{\partial x^{2}}+bW-\left(\sigma_{3}^{0}V+\sigma_{3}^{1}C_{0}\right)V-\sigma_{3}^{1}V_{0}J\left(C\right),$$

$$\frac{\partial C}{\partial t}=kV-\sigma_{4}C.$$

We look for the solution of this system of equations in the following form:

$$W\left(x,t\right)=pe^{i\xi x}e^{\lambda t},\;V\left(x,t\right)=qe^{i\xi x}e^{\lambda t},C\left(x,t\right)=re^{i\xi x}e^{\lambda t}.$$

The eigenvalues *λ* can be found from the equation

(6.1)
det (A−λE)=0,

where

$$A=(\begin{array}{ccc} -\sigma_{2}aU_{0}0b & -\sigma_{3}^{0}-\sigma_{3}^{1}C_{0}-D\xi^{2} & -\sigma_{3}^{1}V_{0}\tilde{\phi }\left(\xi \right)\\ 0 & k & -\sigma_{4} \end{array}),$$

*E* is the identity matrix, and $\tilde{\phi }\left(\xi \right)$ is the Fourier transform of the function *ϕ*(*x*). We have

$$\begin{array}{l} \det\;(A-\lambda E)=-\left(\sigma_{2}+\lambda \right)\left(\sigma_{4}+\lambda \right)\left(\sigma_{3}^{0}+\sigma_{3}^{1}C_{0}+D\xi^{2}+\lambda \right)\\ \;+abU_{0}\left(\sigma_{4}+\lambda \right)-\sigma_{2}k\sigma_{3}^{1}V_{0}\tilde{\phi }\left(\xi \right). \end{array}$$

Set

$$\begin{array}{l} F_{\xi }\left(\lambda \right)=-\left(\sigma_{2}+\lambda \right)\left(\sigma_{4}+\lambda \right)\left(\sigma_{3}^{0}+\sigma_{3}^{1}C_{0}+D\xi^{2}+\lambda \right)\\ \;+abU_{0}\left(\sigma_{4}+\lambda \right) \end{array}.$$

Then equation (6.1) can be written as follows:

(6.2)
$$F_{\xi }\left(\lambda \right)=\sigma_{2}k\sigma_{3}^{1}V_{0}\tilde{\phi }\left(\xi \right).$$

Let us note that *F*_0_(0)=0 and *F*_0_(*λ*)<0 for *λ*>0 . Therefore, if *R*_*v*_>1, then the right-hand side of equation (4.8) for *ξ*=0 is positive ( $\tilde{\phi }\left(0\right)=1$ ), and it cannot have non-negative real solutions. This conclusion is in agreement with stability of the endemic equilibrium for *R*_*v*_>1 . However, if $\tilde{\phi }\left(\xi \right)$ becomes negative for some *ξ* , then equation (6.2) can have a positive solution. Substituting *λ*=0 in this equation (stability boundary), we obtain

$$\tilde{\phi }\left(\xi \right)=-\frac{D\xi^{2}}{\sigma_{3}^{0}\left(R_{0}-1\right)}.$$

If the function *ϕ*(*ξ*) becomes negative for some *ξ* , *R*_*v*_>1 and *D* is sufficiently small, then this equation has a solution. In this case, the homogeneous in space stationary solution loses its stability leading to the emergence of a stationary periodic in space solutions.

## Appendix 2. Numerical implementation

The system of equations (5.1)-(5.6) is solved numerically by the finite difference method with a uniform space grid *x*_*i*_=*idx* (*i*=1,...,*I*) , where *dx* is the space step. The values $U_{i}^{n+1}$ , $V_{1}i^{n+1},V_{2}i^{\mathrm{n+}1}$ , $W_{1}i^{n+1},\text{and }W_{2}i^{n+1}$ at time step *n*+1 are found by the formulas:

$$U_{i}^{n+1}=\frac{U_{i}^{n}+\kappa \Delta t}{1+\Delta t\left(a_{1}V_{1}i^{n}+a_{2}V_{2}i^{n}+\sigma_{1}\right)},$$

$$W_{1}i^{n+1}=\frac{W_{1}i^{n}+a_{1}U_{i}^{n+1}V_{1}i^{n}\Delta t}{1+\Delta t\left(\sigma_{21}^{0}+\sigma_{21}^{1}C^{n}\right)},$$

$$W_{2}i^{n+1}=\frac{W_{2}i^{n}+a_{2}U_{i}^{n+1}V_{2}i^{n}\Delta t}{1+\Delta t\left(\sigma_{22}^{0}+\sigma_{22}^{1}C^{n}\right)}.$$

Equations (5.4) and (5.5) are approximated by a three-point implicit finite difference scheme:

$$\frac{V_{1}i^{n+1}-V_{1}i^{n}}{\Delta t}=D\frac{V_{1}{i+1}^{n+1}-2V_{1}i^{n+1}+V_{1}{i-1}^{n+1}}{{\left(\Delta x\right)}^{2}}+b_{1}W_{1}i^{n+1}-\left(\sigma_{31}^{0}+\sigma_{31}^{1}C^{n}\right)V_{1}i^{n+1}$$

(and similar for the second equation). This equation, together with the homogeneous Neumann boundary condition, is solved by the Thomas algorithm for the inversion of the corresponding tridiagonal matrix. Finally, the equation for the concentration of immune cells is approximated by the following finite difference equation:

$$C^{n+1}=\frac{C^{n}+\left(k_{1}I\left(V_{1}^{n+1}\right)+k_{2}I\left(V_{2}^{n+1}\right)\right)\Delta t}{1+\sigma_{4}\Delta t}\;.$$

Note that each next finite difference equation uses the values from the (n+1)th step found in the previous equations. The order of the equations can be changed. The accuracy of numerical simulations was controlled by decreasing time and space steps and by the comparison with the analytical results when they are available.

Numerical implementation was carried out with programming language Python 3.8 and graphical library matplotlib 3.3.2.

## Appendix 3. The values of parameters

Some values of parameters were determined in (1, 35) by fitting the experimental data in (4, 5) on the Delta and Omicron variants of SARS-CoV-2 infection. In the study of the emergence of new variants, the values of parameters were chosen to provide the instability of the homogeneous in space solution.

**Table:**

Parameter	Meaning	Value	References
a	Cell infection rate (1/hour/virion)	*a* = 0.01 (10^−4^ - 0.1)	(1, 35, 36)
b	Virus replication rate (virion/cell/hour)	*b* = 10^3^ − 10^6^	(1, 35, 36)
k	Stim. of immune cells by antigen (1/virion/hour)	k = 10−^6^ − 10−^5^	ETW (*)
*κ*	Influx of epithelial cells (cell/hour)	0.001 - 0.01	(37)
*σ*_1_	Death rate of uninfected cells (1/hour)	0.001	(37)
$\sigma_{2}^{0}$	Death rate of infected cells (1/hour)	0.001 - 0.1	(1, 35, 36)
$\sigma_{2}^{1}$	Cell elimination by imm. response (1/cell/hour)	0.1	ETW
$\sigma_{3}^{0}$	Virus death rate (1/hour)	0.1 - 1	(1, 35, 36)
$\sigma_{3}^{1}$	Virus elimination by imm. response (1/cell/hour)	1	ETW
*σ*4	Death rate of immune cells (1/hour)	**0.01-0.01**	(38)

(*) ETW - estimate/this work.

Bold values indicated death rate of infected cells.

Sensitivity analysis is carried out in a recent work (39) for a more complete model with the innate and adaptive immune responses (different questions were investigated).
